# Comprehensive characterization of immunogenic cell death in acute myeloid leukemia revealing the association with prognosis and tumor immune microenvironment

**DOI:** 10.1186/s12920-024-01876-w

**Published:** 2024-04-26

**Authors:** Yongyu Chen, Xue Qiu, Rongrong Liu

**Affiliations:** 1 Department of Hematology, The first Affiliated Hospital of Guangxi Medical University, Nanning, China; 2 Department of Cardiology, The first Affiliated Hospital of Guangxi Medical University, Nanning, China; 3 Guangxi Medical University, Nanning, China

**Keywords:** Acute myeloid leukemia, Immunogenic cell death, Tumor microenvironment, Clinical application

## Abstract

**Background:**

This study aimed to explore the clinical significance of immunogenic cell death (ICD) in acute myeloid leukemia (AML) and its relationship with the tumor immune microenvironment characteristics. It also aimed to provide a potential perspective for bridging the pathogenesis of AML and immunological research, and to provide a theoretical basis for precise individualized treatment of AML patients.

**Methods:**

Firstly, we identified two subtypes associated with ICD by consensus clustering and explored the biological enrichment pathways, somatic mutations, and tumor microenvironment landscape between the ICD subtypes. Additionally, we developed and validated a prognostic model associated with ICD-related genes. Finally, we conducted a preliminary exploration of the construction of disease regulatory networks and prediction of small molecule drugs based on five signature genes.

**Results:**

Differentially expressed ICD-related genes can distinguish AML into subgroups with significant differences in clinical characteristics and survival prognosis. The relationship between the ICD- high subgroup and the immune microenvironment was tight, showing significant enrichment in immune-related pathways such as antibody production in the intestinal immune environment, allograft rejection, and Leishmaniasis infection. Additionally, the ICD- high subtype showed significant upregulation in a variety of immune cells such as B_cells, Macrophages_M2, Monocytes, and T_cells_CD4. We constructed a prognostic risk feature based on five signature genes (TNF, CXCR3, CD4, PIK3CA and CALR), and the time-dependent ROC curve confirmed the high accuracy in predicting the clinical outcomes.

**Conclusion:**

There is a strong close relationship between the ICD- high subgroup and the immune microenvironment. Immunogenicity-related genes have the potential to be a prognostic biomarker for AML.

**Supplementary Information:**

The online version contains supplementary material available at 10.1186/s12920-024-01876-w.

## Introduction

Acute myeloid leukemia (AML) is a common blood disease which derived from abnormal growth of bone marrow cells, which has a high degree of specificity in human populations [1, 2], and the research on the pathogenesis of AML has not yet been fully clarified [3]. With the improvement of chemotherapy, hematopoietic stem cell transplantation and other treatments, the prognosis of AML patients has been improved to a certain extent [4, 5], but in general, AML is characterized by poor overall efficacy, high recurrence rate and poor long-term survival prognosis, and there is a long way to go for the long-term survival of AML patients [6].

Immunogenic death (ICD) is one of the modes of regulatory cell death, and ICD can induce adaptive immunity against dead cell antigens by mediating the complex connection between the immune system and dead tumor cells, thus improving the sensitivity and efficacy of immunotherapy [7, 8]. Immunogenicity-related genes play a prominent role in cancer. These genes encode the proteins required by the immune system in the body, including antigen-presenting molecules, lymphocyte receptors, and cytotoxic proteins. By regulating the expression level of these immunogenic genes, the immune system can recognize and eliminate abnormal cells, thereby preventing the occurrence and development of cancer. Hence, investigating the role of immunogenic genes in cancer is of great significance for understanding the interaction between the immune system and cancer, and developing immunotherapy strategies. By regulating the expression of immunogenic genes, the immune system’s ability to attack cancer cells can be enhanced, and thus treatment effectiveness can be improved [9]. As a specific form of cell death that activates tumor-specific immune responses to exert anti-tumor effects, immunogenic cell death has been a hot topic in oncology research in recent years [10, 11]. However, there is limited in-depth research on ICD-related genes (ICDGs) in AML.

In recent years, the rapid development of high-throughput sequencing technology has greatly facilitated the in-depth exploration of disease characteristics, pathogenesis, and risk stratification while providing a scientific basis for individualized treatment, therapeutic efficacy and prognostic judgment of patients [3, 12, 13] .Therefore, we intend to comprehensively investigate the clinical significance of immunogenic death in AML and its relationship with tumor immune microenvironment characteristics, to provide a novel perspective for in-depth pathogenesis and immunobiological studies of AML, and to provide a theoretical basis for the precise treatment and management of AML patients through the construction of a prognostic prediction model related to immunogenic death.

## Materials and methods

### Data source and access

For training cohort, RNA-seq transcriptome information and matched baseline data were obtained from 151 AML patients from TCGA database (https://portal.gdc.cancer.gov/). Patient data with complete transcription and clinical information from dataset GSE37642 in the Gene Expression Omnibus (GEO) database (https://www.ncbi.nlm.nih.gov/geo/) were downloaded and included in the testing cohort. All eligible samples from TCGA were collected according to the following inclusive criteria: (1) diagnosed AML specimen; (2) availability of transcriptome data; and (3) availability of general survival information and related clinical data. A total of 130 bone marrow samples on TCGA database were included in this study. The detailed pre-processing of microarray data of GSE37642 cohort was illustrated below: (1) The samples that lacked of corresponding follow-up information were eliminated; (2) The Gene Symbol format was obtained by converting the probe IDs; (3) Probes were removed because of their correspondences to multiple genes; (4) The average value was regarded as the gene expression while multiple probes were corresponded to one gene. In final, 140 bone marrow samples on GSE37642 cohort were included.

### ICD classification through consensus clustering analysis

We used the Consensus Cluster Plus tool for clustering analysis through Sangerbox website (http://vip.sangerbox.com) to identify molecular subtypes related to ICD. Subsequently, the optimal number of clusters was determined using empirical cumulative distribution function plots to ensure stable results. Finally, we use the heatmap tool to create cluster maps.

### Identification of differentially expressed genes (DEGs)

Linear Models for Microarray Data (Limma) is a package in R language used for analyzing microarray data, including RNA Seq and other high-throughput sequencing data. It provides a series of powerful statistical models and methods for identifying differentially expressed genes, conducting common expression data analysis and visualization. In the study, the “limma” programme was utilized through Sangerbox website to determine the DEGs between C1 and C2 subtypes (by comparing different clusters), and the filtering thresholds were adjusted *P* < 0.05 and |log2 Fold change| > 2.

### Functional enrichment analysis

The functional enrichment analysis was performed by Gene Ontology (GO) and Kyoto Encyclopedia of Genes and Genomes (KEGG) databases, for the aim of comparing the differential signal pathway and biological effects among the ICD low and high cohorts. The P-value thresholds was 0.05. Gene set enrichment analysis (GSEA) was conducted to assess whether there were considerable variations in the set of genes expressed between the ICD low and high cohorts in the enrichment of the MSigDB Collection (c2.cp.kegg.v7.4.symbols.gmt), a P-value < 0.05 and FDR < 0.25 were considered statistically significant differences.

### Somatic mutation analysis and characterization of the immune landscape between two ICD subgroups

Somatic mutation data of AML samples were obtained from the TCGA database, and the specific differences in somatic mutation data between two ICD subgroups samples were presented in waterfall plots through Sangerbox website. To analyzed immune cell infiltration and explore TME characteristics between the two ICD subgroups, immune infiltration analysis by multiple algorithms (XCell、quanTlseq、MCPCOUNTER and CIBERSORT) were conducted in the training and validation sets through online analysis websites (http://www.sxdyc.com).

### Construction of ICD-related risk features

Least absolute shrinkage and selection operator (LASSO) is a commonly used regression analysis method that combines variable selection and regularization to improve the predictive performance and interpretability of the resulting statistical model. In this study LASSO regression analysis was used to identify independent risk factors for AML. The R programming language “RMS” was used to create a prognostic nomogram to determine its value in prognostic prediction. Subsequently, K-M survival analysis was performed to explore the feasibility of constructing the risk model under different clinical features.

### Construction of miRNA-gene, TF-gene regulatory networks, identification of potential targeted drugs and drug sensitivity analysis

Networkanalyst software (https://www.networkanalyst.ca/) was used to predict miRNAs and transcription factors that interact with ICD-related genes, respectively, and to construct regulatory networks to predict the interactions between miRNAs and genes, as well as TFs and genes. The Drug-Gene Interaction database (https://dgidb.org/) was used to search for potential targeted drugs and attempt to find the relationship between drug-gene interactions. Considering that a great number of patients with acute myeloid leukemia have poor prognosis and may experience disease recurrence, drug resistance, and other conditions throughout the treatment process, we conducted a drug sensitivity analysis in this study, aiming to provide more potential treatment strategies for the vast number of acute myeloid leukemia patients. The drug sensitivity analysis was conducted from cellminer database (https://discover.nci.nih.gov/SclcCellMinerCDB/) and other comprehensive drug screening datasets (https://zenodo.org/records/7274740 and https://biodev.github.io/BeatAML2).

### Single-cell expression analysis and subcellular localization of biomarkers

On the basis of the HPA database (https://www.proteinatlas.org/), single-cell data and transcriptional data were utilized to assess the expression of biomarkers in bone marrow cells. Based on the COMPARTMENTS database (https://compartments.jensenlab.org/), we also predicted biomarker protein subcellular localization. This website serves as a prediction tool for proteins’ subcellular locations.

### Statistical analysis

Comparison of continuous variables between two groups was performed using two independent samples t-test, and chi-square test was used for categorical data. The Kaplan–Meier method and log-rank test were used to estimate patient survival and plot survival curves. P value less than 0.05 was considered statistically significant.

**Fig. 1:**
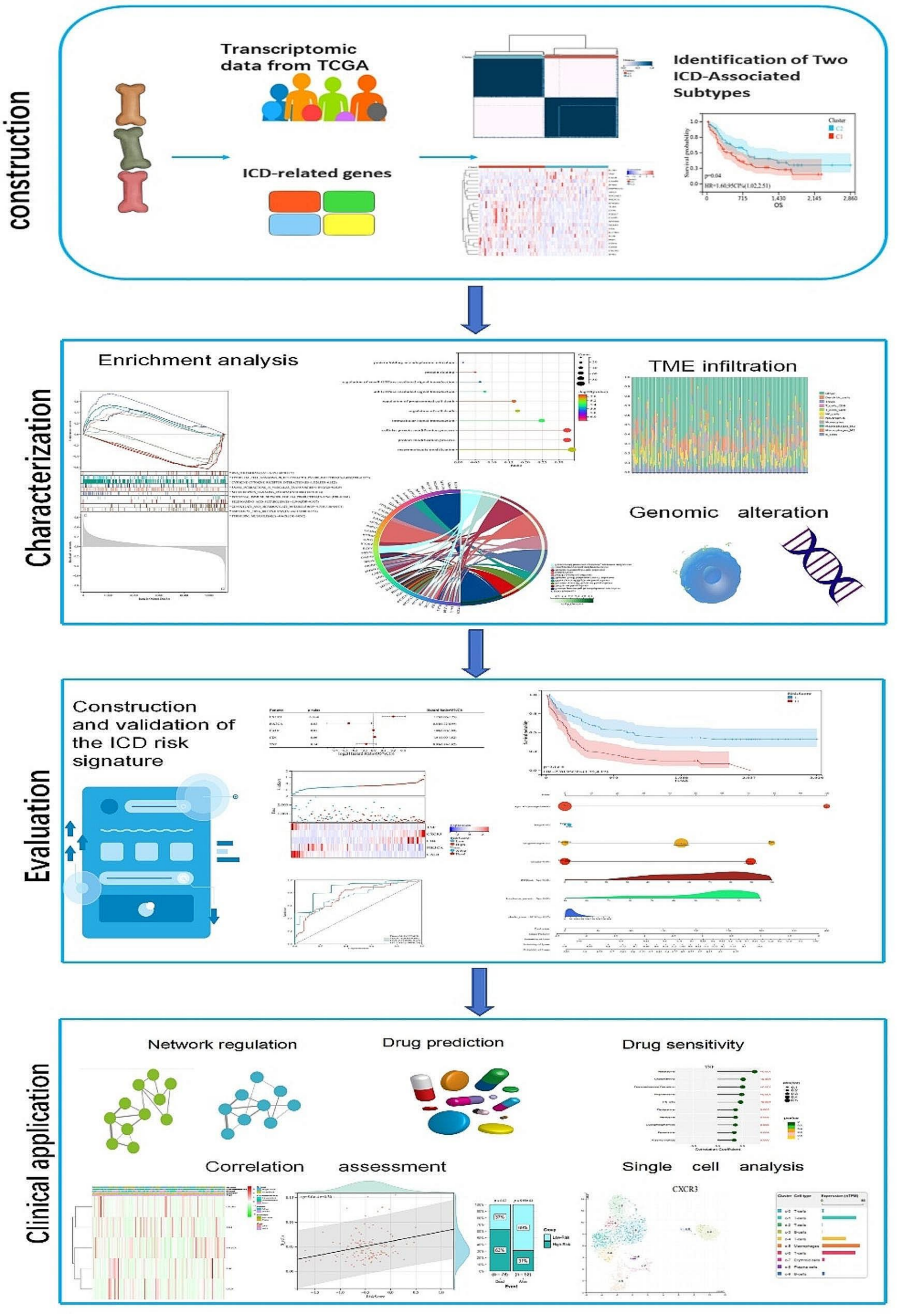
Flow chart of the study

The research process for this article is shown in Fig. 1.

## Result

### Identification of two ICD-related subtypes by consensus clustering

**Fig. 2:**
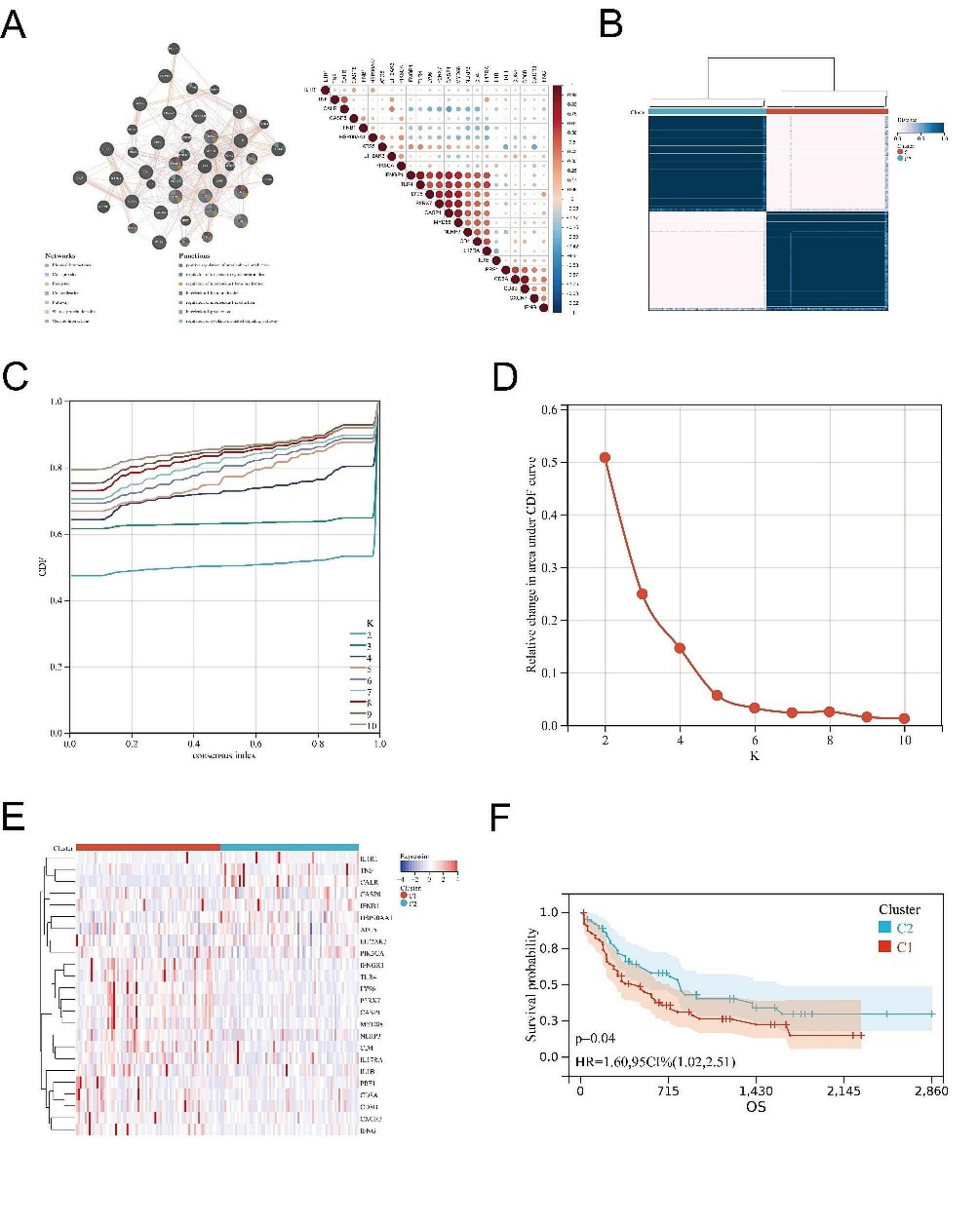
Identification of ICD-associated subtypes by consensus clustering. (A) Construction of interactive functions between ICD-related genes; (B) Identification of two ICD-related subtypes by consensus clustering; (C, D) The delta area curve of consensus clusters represents the relative change of the area under the cumulative distribution function (CDF) curve from k = 2 to 10; (E) Heatmap of the expression of ICD-related genes in different subtypes. Red represents high expression; blue represents low expression; (F) Kaplan-Meier curves for different ICD subtypes

We identified ICD-related genes (TNF, CXCR3, P2RX7, CASP1, NLRP3, IL1B, LY96, CD4, CD8A, CD8B, PRF1, IFNG, IL17RA, HSP90AA1, EIF2AK3, PIK3CA, CASP8, ATG5 IL1R1, MYD88, IFNGR1, CALR, TLR4) by summarizing the studies of Garg AD et al. [14] and we used the genemania database (http://genemania.org/) to reveal the interactions among these ICD-related genes (Fig. 2A). Next, we used consensus clustering analysis to determine the AML clusters related to ICD, where we divided the samples into two clusters (Fig. 2B-D). Then, we constructed a heatmap of the expression of ICD-related genes in the C1 and C2 clusters (Fig. 2E). Survival analysis showed that different expressions of ICD-related genes led to statistically significant discrepancies in survival outcomes among subgroups (Fig. 2F).

### Enrichment analysis and identification of signaling pathways

**Fig. 3:**
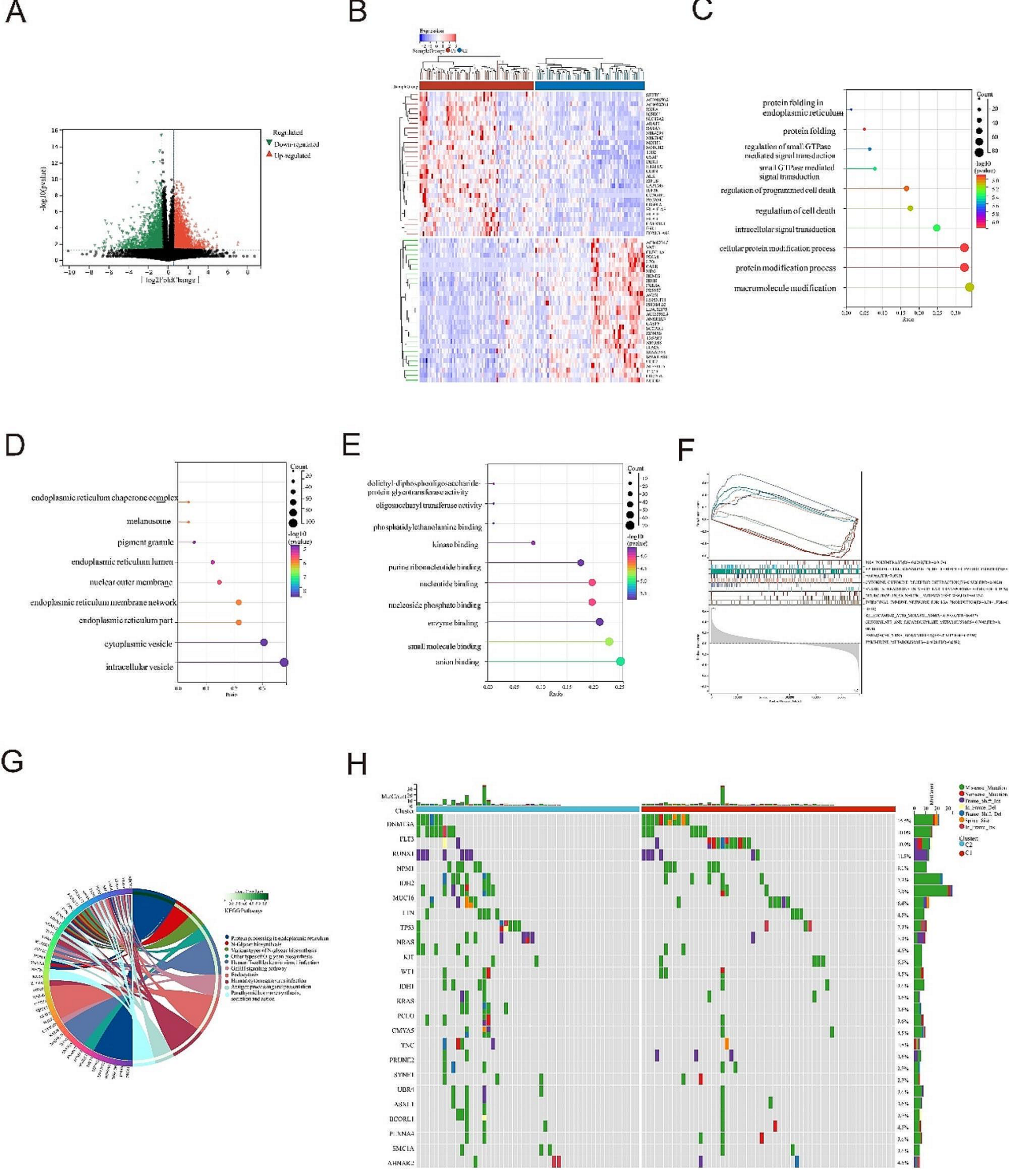
Identification of differentially expressed genes (DEGs) and potential signaling pathways in the subtypes. (A) Volcano plot showing the quantified DEG distribution between the ICD-high and the ICD-low subtype with threshold of |log2 Fold change| > 2 and *P* < 0.05 in TCGA cohort; (B) Heatmap shows the expression of DEG in different subtypes; (C, D, E) Biological Process, Cellular Component and Molecular Function in GO signaling pathway enrichment analysis. The size of dots represents gene counts and the color of dots represents– log10 (p. adjust-value); (F) GSEA analysis identifies potential signaling pathways between subtypes; (G) KEGG pathway analysis; (H) Comparison of somatic mutations between subtypes

We identified key genes and signaling pathways in subtypes to understand the molecular mechanisms associated with prognosis. There are 2,847 genes that differ significantly between ICD subgroups (*p* < 0.05), including 1577 up-regulated genes and 1270 down-regulated genes (Fig. 3A). The top 50 significantly expressed genes were visualized in a heatmap (Fig. 3B). We then attempted to understand the molecular mechanisms underlying the regulation of disease prognosis. In Fig. 3C-E, functional enrichment analyses were performed to determine the biological classification, function, and pathway of DEGs. GO analysis revealed that in the biological processes category(Fig. 3C), DEGs were enriched in protein folding in endoplasmic reticulum, regulation of small GTPase mediated signal transduction, regulation of cell death, cellular protein modification process and protein modification process. Meanwhile, in the cellular component category (Fig. 3D), DEGs were enriched in endoplasmic reticulum lumen, nuclear outer membrane, endoplasmic reticulum part, cytoplasmic vesicle, intracellular vesicle and endomembrane system. In the molecular function category (Fig. 3E), DEGs were enriched in oligosaccharyl transferase activity, kinase binding, purine ribonucleotide binding, nucleotide binding, nueleoside phiosphate binding, enzyme binding and small molecule binding activity. GSEA results showed that in ICD-high subgroup (C1 cluster), immune-related pathways such as antibody production in the intestinal immune environment, allograft rejection and leishmaniasis infection were significantly enriched. On the other hand, in ICD-low subgroup (C2 cluster), pathways related to genetic replication and biomolecular synthesis were significantly enriched (Fig. 3F). KEGG enrichment analysis revealed functional pathways related to protein synthesis and processing, polysaccharide biosynthesis, leukemia virus infection and antigen processing and presentation in human cytomegalovirus infection, among other variable expression genes (Fig. 3G).

### Somatic mutations and tumor microenvironment landscapes in ICD subtypes

**Fig. 4:**
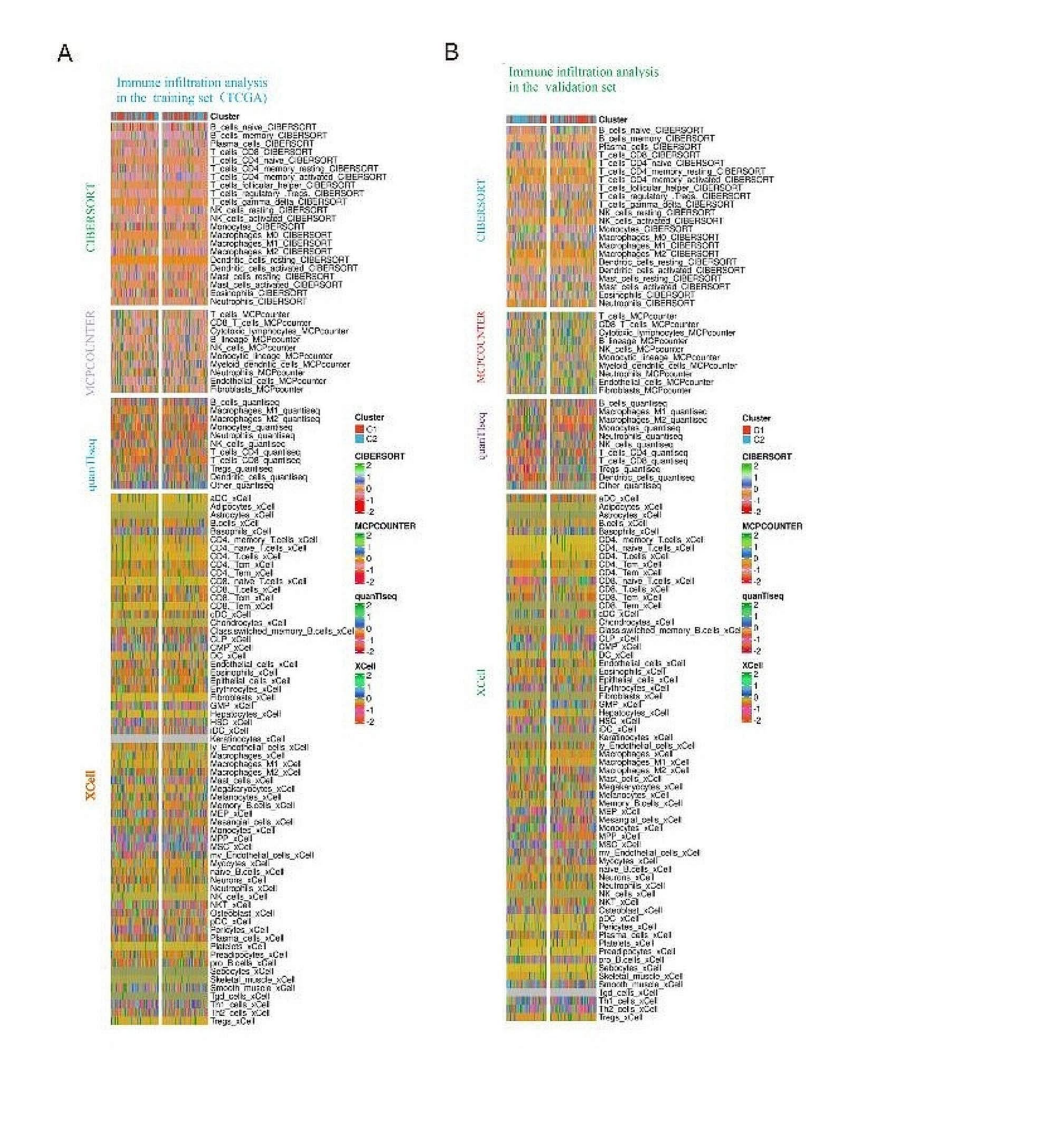
Immune landscape between ICD subtypes. (A) Immune infiltration analysis by multiple algorithms in the training set; (B) Immune infiltration analysis by multiple algorithms in the validation set

We found distinct somatic mutation profiles in ICD subtypes and observed that gene mutations such as DNMT3A, FLT3, RUNX1, and NPM1 occurred at higher frequencies in AML patients’ somatic mutations. However, their relative frequencies differed between subgroups (Fig. 3H). In view of the important biological role of ICD in antitumor immune response, the microenvironment of tumors between subgroups was carefully studied by multiple algorithms (XCell、quanTlseq、MCPCOUNTER and CIBERSORT) in the training and validation sets. We compared the expression of different immune cells between subgroups and found that B_cells, Macrophages_M2, Monocytes, and T_cells_CD4 were significantly upregulated in the ICD- high subtype (C1 cluster), while NK_cells were significantly downregulated (Fig. 4A-B). The above results exposed the close relationship between ICD profile and tumor microenvironment.

### Construction and validation of ICD risk signature

**Fig. 5:**
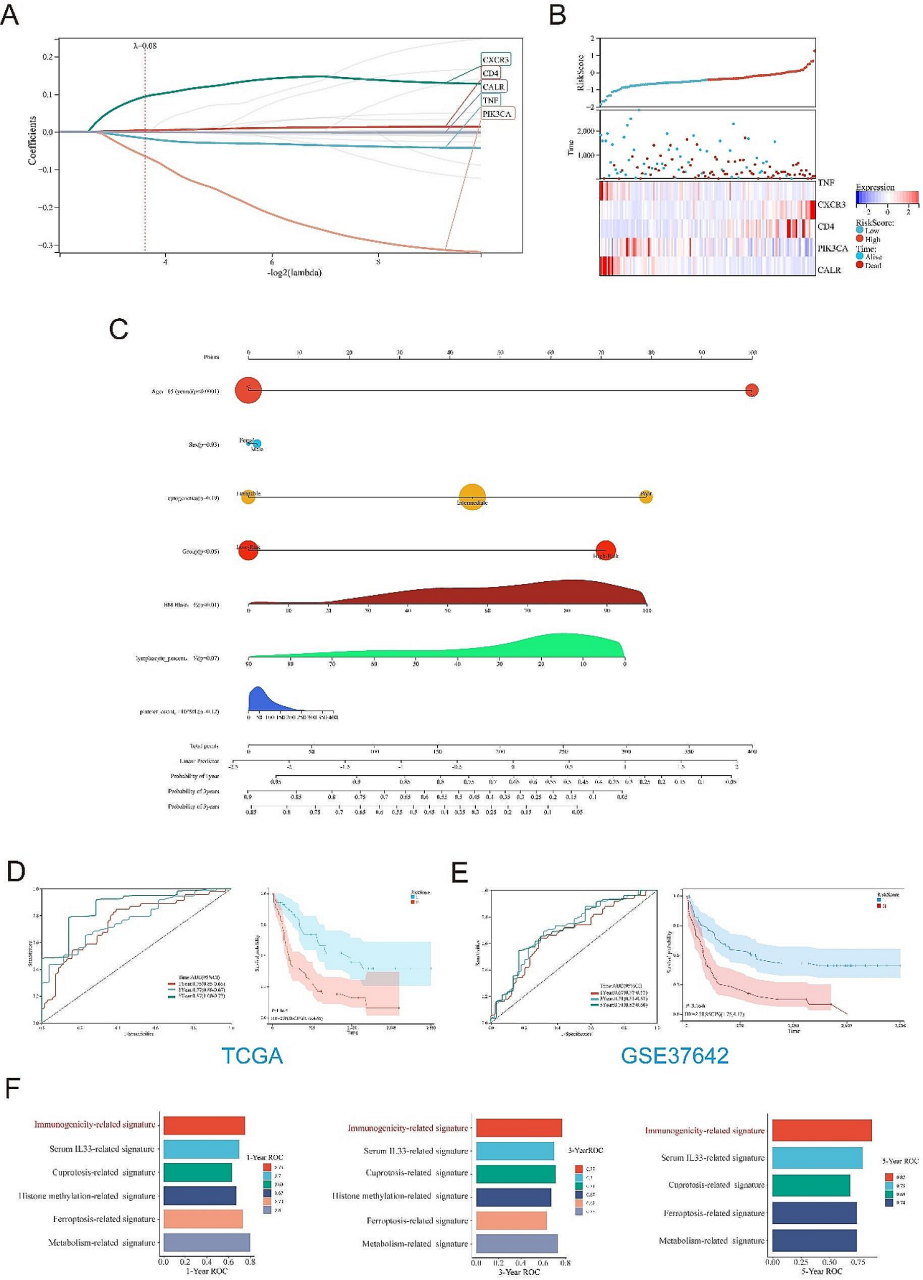
Construction and validation of the ICD-risk signature. (A) Lasso analysis identified five genes most related to prognosis in TCGA dataset; (B) Risk scores distribution, survival status of each patient, and heatmaps of prognostic 5-gene signature in TCGA database; (C) Construction of the nomogram; Time-dependent ROC curves and Kaplan-Meier analysis in the TCGA cohort (D) and GSE37642 cohort (E). (F) Performance comparison between ICD-risk signature and other signatures based on receiver operating characteristic curve

Five prognostic genes (TNF, CXCR3, CD4, PIK3CA and CALR) were detected as predictive model-related genes in LASSO regression analysis (Fig. 5A). Figure 5B showed the relationship between risk scores and survival status, overall, the low-risk cohort had more survivors than the high-risk cohort. We found that the nomogram constructed by risk scores and patients’ clinical data showed higher predictive value compared to most clinical information (Fig. 5C). Next, we used ROC and Kaplan Meier curves to evaluate the prognostic ability of these 5 ICD-related genes. Time-dependent ROC curves indicated good predictive performance of the model (AUC for training cohort at 1 year, 3 years, and 5 years were 0.75, 0.77 and 0.87, respectively; while AUC for testing cohort at 1 year, 3 years, and 5 years were 0.67, 0.71 and 0.71, respectively) (Fig. 5D-E). Compared with several other published signatures and popular biomarkers, ICD-related signature had the highest AUC for either 3-year or 5-year survival (Fig. 5F).

### Clinical application of ICD-risk signature

**Fig. 6:**
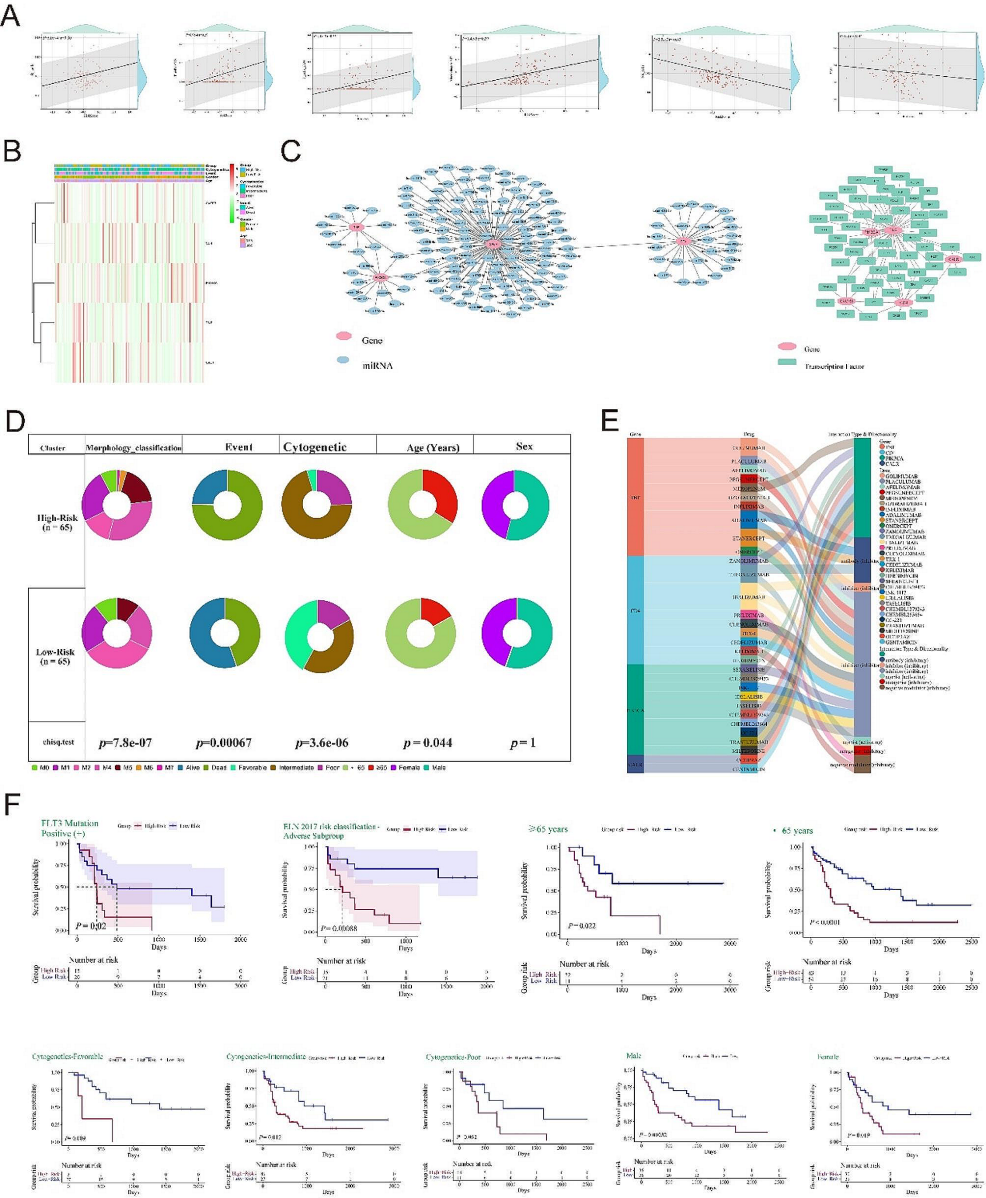
Clinical application of the ICD-risk scores. (A) Correlation analysis of ICD- risk scores and expression of various types of immune cells (B cell, CD8 T cell, CD4 T cell, Macrophages-M2, NK cell and Treg cells); (B) Correlation of ICD-risk genes and clinical information; (C) miRNA-gene and transcription factor-gene regulatory network was constructed to initially explore the pathogenesis of acute myeloid leukemia; (D) Different comparisons of number of patients in morphology classification, age, sex, cytogenetics and survival events between subgroups; (E) Identification of the targeted drugs for ICD prognosis genes; (F) Subgroup survival analysis

To evaluate the utility of constructing the ICD risk signature, we firstly analyzed the correlation between risk scores and the expression levels of immune cells. Interestingly, we found a significant positive correlation between the expression levels of immune cells such as B_cells, T_cells_CD8, T_cells_CD4, Macrophages_M2 and the risk scores, while the expression levels of immune cells such as NK_cells, cells_Treg showed an opposite trend with the risk scores (Fig. 6A). Next, we generated a complex heatmap to visualize the correlation between the expression levels of ICD risk genes and clinical information (Fig. 6B). We then compared the differences in the number of individuals between subgroups in terms of clinical data such as morphology classification, age, gender, cytogenetics and survival events (Fig. 6D) in order to demonstrate the successful construction of the ICD risk signature and its value for clinical application. We finally conducted subgroup survival analysis on patients with FLT3 mutation positive, adverse outcome in EIN 2017 risk stratification and other parameters (gender, age et al.) based on ICD-signature to verify the feasibility of this prediction model. (Fig. 6F).

### Construction of regulatory networks, prediction of small molecule drugs and drug sensitivity analysis

**Fig. 7:**
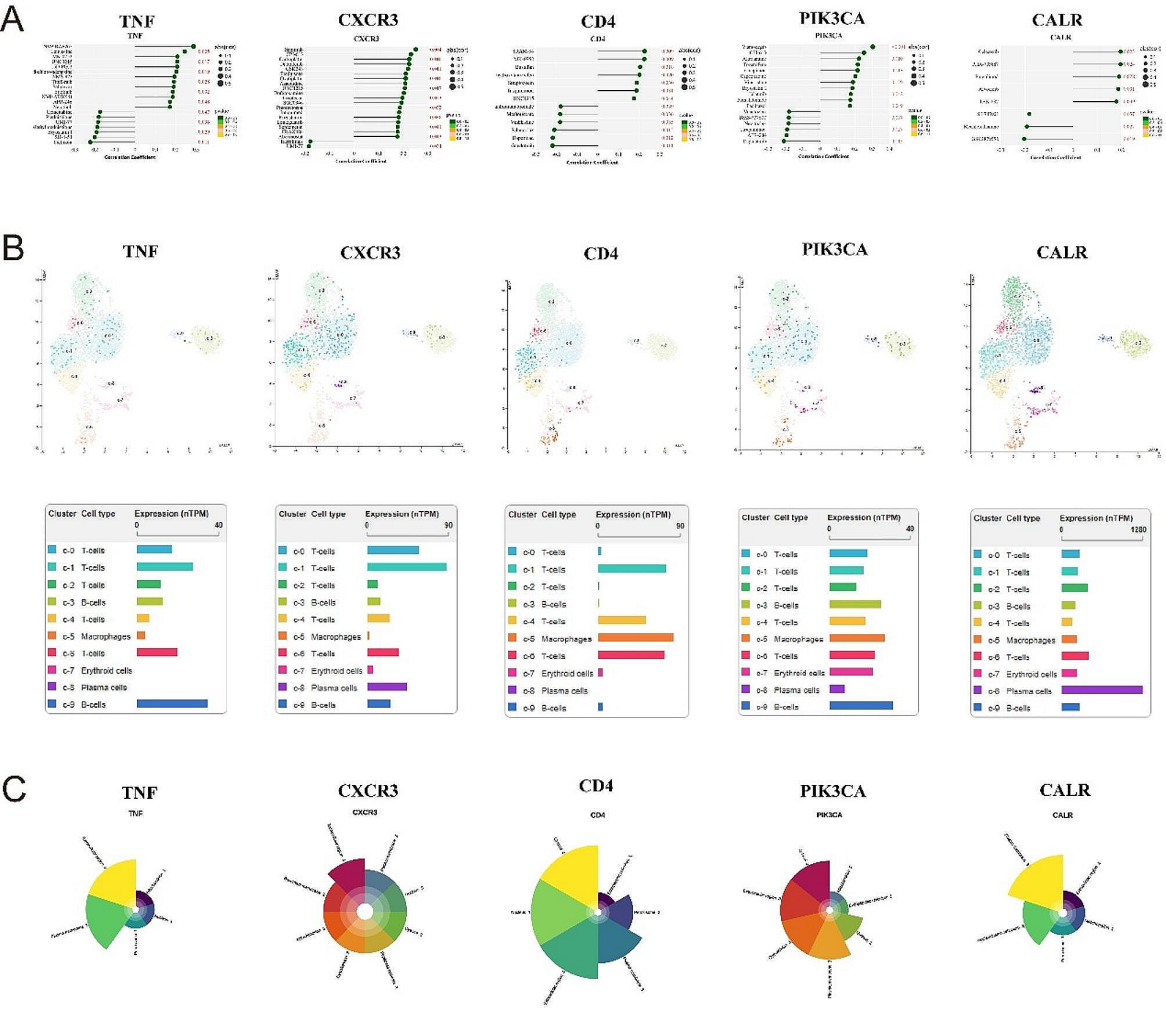
(A) Drug sensitivity analysis of ICD prognosis genes; Single-cell expression analysis (B) and subcellular localization analysis (C) of hub genes

Transcription factors (TFs) can bind to specific DNA sequences and regulate gene expression under specific conditions, while microRNAs (miRNAs) are a class of endogenous short non-coding RNAs that can effectively mediate mRNA degradation. To gain a deeper understanding of the pathogenesis of AML, we used NetworkAnalyst software to predict the transcription factors and miRNAs that regulate ICD genes, and constructed transcription factor-gene and miRNA-gene regulatory networks. There are a total of 61 TFs and 157 miRNAs that exhibit complex regulatory relationships with ICD-signature genes (Fig. 6C). Meanwhile, we used the DGIdb database (Drug-Gene Interaction database) to retrieve targeted drugs for ICD genes and the drug-gene interactions, with the hope of providing theoretical reference for precise treatment of AML (Fig. 6E). At the same time, the correlation between drug score and prognostic genes was analyzed, and the research results showed that chemotherapy drugs such as Lomustine. Tipifarnib, Selenexor. etc. exhibited good therapeutic effects on the TNF gene. For the CXCR3 gene, chemotherapy drugs such as Sapitinib, Carboplatin, and Dabrafenib were more sensitive to it and had shown therapeutic potential. For the CD4 gene, potential therapeutic drugs such as TRAM-34 and Streptozocin had shown sensitive therapeutic effects, For the PlK3CA gene. chemotherapy drugs such as Vismodegib, CPI-613, and Altretamine were more sensitive to it. While the commonly used chemotherapy drug venetoclax in clinical practice has poor response to the treatment of the PlK3CA gene. For the CALR gene, potential therapeutic drugs such as Celecoxib, PHA-793,887, and Fingolimod were more sensitive to it. Overall, the above findings were conducive to providing potential treatment strategies for AML patients (Fig. 7A).

### Single cell analysis and subcellular localization of prognostic genes

To more accurately describe the expression of prognostic genes in human bone marrow tissue, we applied scRNA seq based on the HPA database to identify the cell populations expressed in the bone marrow. Nine subpopulations of bone marrow cells were identified through clustering, as shown in the UMAP plot. The results further revealed the main expression of TNF in T cells and B cells, CXCR3 was mainly expressed in macrophages and T cells and CD4 was mainly expressed in T cells, B cells and plasma cells. The main expression of PIK3CA in macrophages, T cells, and B cells as well as the main expression of CALR in plasma cells (Fig. 7B).

Proteins mark different biological functions, depending on their position in the cell. Based on the Compartments database, we further predicted the protein subcellular localization of prognostic genes. TNF was mainly distributed on the plasma membrane, CXCR3 was mainly distributed outside the cell and in the plasma membrane, CD4 was mainly distributed on the plasma membrane, PIK3CA was mainly distributed on the cytoskeleton and in the plasma membrane, and CALR was mainly distributed on the cytoplasmic reticulum and in the plasma membrane (Fig. 7C).

### GSEA of biomarkers and expressions of the five genes of the prognostic signature in acute myeloid leukemia

**Fig. 8:**
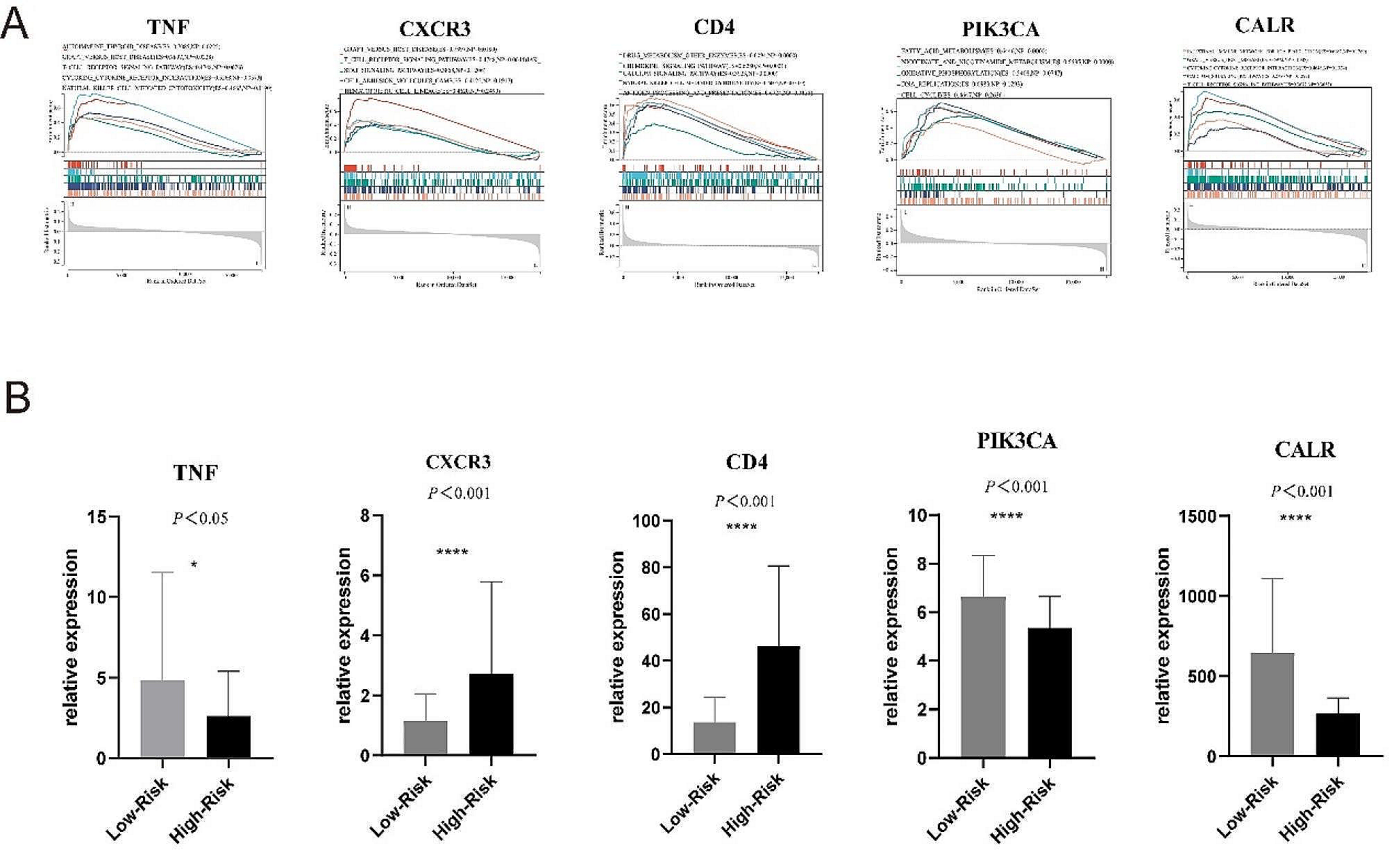
(A) Gene set enrichment analysis for the five-gene signature; (B)Expressions of the five genes of the prognostic signature in acute myeloid leukemia

In the end, we used the GSEA to perform GO items to explore the possible roles of the chosen biomarkers in AML. As shown in Fig. 8A, high- expression group of the 5 ICD-prognosis genes of the prognostic signature was mainly enriched to immune-related pathways. The above results indicated the close and complex relationship between the ICD prognosis model and the immune system, which provided preliminary directions for further exploring the pathogenesis of AML in the future. Finally, expression levels of the five genes of the prognostic signature in acute myeloid leukemia were depicted in Fig. 8B.

## Discussion

Acute myeloid leukemia is a common type of acute leukemia, which is characterized by poor overall efficacy, high relapse rate and poor long-term survival prognosis [15]. Currently, the pathogenesis of AML is still unexplored, and accurate assessment of the prognosis of patients and appropriate treatment thus become needed. Therefore, it is necessary to construct a reasonable prognostic assessment model. Immunogenic cell death is a complex process driven by stress, involving various signaling molecules and cytokines, which can trigger a complete antigen-specific adaptive immune response by releasing danger signals or damage-associated molecular patterns (DAMPs) [16]. In-depth study of the molecular mechanisms of immunogenic cell death in leukemia is beneficial for providing new insights into the comprehensive treatment of the disease. Therefore, we have screened ICD-related genes to construct an AML prognostic assessment model and attempt to explore its possible molecular mechanisms and clinical applications.

We firstly determined two ICD subgroups, Cluster 1 and Cluster 2, based on consensus clustering using ICD-related gene expression. There were significant differences in clinical prognosis and immune infiltration levels between the ICD subgroups. The C1 subtype with higher ICD immune scores was associated with numerous immune functional pathways, while the C2 subtype with lower ICD immune scores was closely linked to pathways such as genetic replication and biomolecule synthesis. We then attempted to compare the tumor immune microenvironment between different subtypes and identify potential mechanisms of AML pathogenesis and investigate the reasons for prognostic differences between subtypes. Our research results showed that the C1 subtype with higher ICD scores had a higher proportion of immune cell infiltration (such as B cells, CD4 + T cells, macrophages and myeloid dendritic cells). The expression levels of immune cells such as B_cellsT_cells_CD8, T_cells_CD4, and Macrophages_M2 were significantly positively correlated with risk scores, while the expression levels of immune cells such as NK_cells and cells_Treg were significantly negatively correlated with risk scores. Based on our analysis of immune cell infiltration and previous literatures, we speculate that tumor cells can reshape the tumor microenvironment through several mechanisms. On the one hand, tumor cells can effectively evade immune surveillance by reducing the number of T_cells and B_cells [17]. On the other hand, tumor cells weaken NK_cell activity, further inducing immune suppression in patients, ultimately leading to tumor progression and reshaping of the tumor microenvironment [18, 19]. In summary, through the grouping of ICD, we have demonstrated the close correlation between the ICD- high subgroup and the immune microenvironment, which may provide more accurate reference value for future immunotherapy in AML patients.

Gene mutations play a key role in the occurrence, progression, and treatment response of acute myeloid leukemia. A deeper understanding of gene mutations helps to more accurately predict disease progression and prognosis, and provides potential targets for developing new treatment strategies. Currently, it is widely believed that gene mutations mainly affect signaling pathways and hematopoietic regulatory transcription factors, ultimately leading to blockage of hematopoietic cell differentiation and subsequent inhibition of apoptosis, resulting in the occurrence of acute leukemia. In this study, through the construction of an immunogenicity-related prediction model, we found that the occurrence frequency of gene mutations such as DNMT3A, FLT3, RUNX1, and NPM1 was relatively high. Based on published research results, we briefly summarized the impact of these genes on disease occurrence. DNMT3A is a DNA methyltransferase that plays a crucial role in maintaining hematopoietic stem cell homeostasis and promoting hematopoietic cell differentiation. Mutations in DNMT3A can significantly alter genome methylation levels, leading to changes in gene expression and epigenetic regulatory patterns [20], causing blockage of hematopoietic cell differentiation and excessive proliferation. In addition, DNMT3A mutations can also regulate the activation of mammalian target of rapamycin, mTOR, by affecting DNA methylation modifications, thereby influencing the expression of the key cell cycle protein CDK1 and promoting hematopoietic cell proliferation. FLT3 is a receptor tyrosine kinase that plays a crucial role in hematopoiesis and lymphocyte proliferation. Abnormal activation of FLT3 in acute myeloid leukemia is closely associated with disease occurrence and development. Approximately one-third of newly diagnosed AML patients have FLT3 activating mutations, which are associated with poor prognosis [21]. Mutations in the FLT3 gene lead to overactive tyrosine kinase, promoting the growth and division of cancer cells [22]. RUNX1 is a transcription factor that plays a key role in hematopoietic cell differentiation and myeloid development. On one hand, RUNX1 is part of the AML1-ETO fusion protein, which can directly inhibit the transcription of tumor suppressor genes dependent on RUNX1, disrupting normal hematopoietic cell differentiation and promoting leukemia development. On the other hand, RUNX1 can inhibit the activity of other hematopoietic transcription factors such as PU1, GATA1, CEBPA, further disrupting normal hematopoiesis [23]. NPM1 gene mutations play an important role in acute myeloid leukemia. NPM1 mutations result in a stronger nuclear export signal than the nuclear localization signal, leading to abnormal cytoplasmic localization of the mutated NPM1 protein, which is considered to play a crucial role in leukemia occurrence [24].

To specifically quantify the prognostic risk profile of ICD-related genes in AML, we constructed a prognostic risk score composed of five feature genes (TNF, CXCR3, CD4, PIK3CA and CALR). The results showed that the risk score derived from these five ICD-related genes could serve as an independent predictive factor, and patients in the high-risk subgroup had a poorer prognosis. The constructed Nomogram based on this had good predictive value, and the time-dependent ROC curve also confirmed the high accuracy of the risk score in predicting the clinical outcomes of leukemia patients. At the same time, GSEA results revealed that prognostic genes were similarly relevant for disorders in immune signaling pathways, which provide insights for further exploration of the molecular mechanisms and disease management of AML.

We then attempted to achieve a preliminary exploration of the pathogenesis of leukemia through the study of five prognostic genes (TNF, CXCR3, CD4, PIK3CA and CALR). It has been reported that TNF-α is highly expressed in leukemia stem cells (LSC), and its abundant expression is closely associated with poor clinical indicators. In vitro studies have shown that knockout of TNF-α (+) expression can make leukemia cells more sensitive to chemotherapy and delay leukemia relapse (in mouse models) [25]. CXCR3 is expressed on many effector cells in tumors, including CD4 + and CD8 + T cells and natural killer cells. Researcher from the University of California, Berkeley have elucidated the key role of Treg cell expression of CXCR3 in promoting cancer progression, revealing the mechanism by which CXCR3 targeting Treg promotes anti-tumor CD8 + T cell activity, and emphasizing that CXCR3 + Treg is a therapeutic target for cancer immunotherapy [26]. CD4 + T cells are central participants and coordinators of innate and antigen-specific immune responses. Moreover, they are also considered to be anti-tumor effector cells. The balance of pro-tumor and anti-tumor functions of CD4 + T cells largely determines the immunogenicity of the tumor microenvironment [27]. PIK3CA is an oncogene expressed in various organs of normal individuals, such as the brain and digestive tract. PIK3CA mainly plays a role in regulating the proliferation and differentiation of somatic cells. In most cases, this gene is in an inactive state. Once the gene undergoes mutation, PIK3CA is abnormally activated, leading to excessive protein expression and cell transformation [28]. Calcium-binding protein (CALR) is a companion of the endoplasmic reticulum (ER) lumen, usually maintaining the homeostasis of the ER by acting as a Ca2 + buffer and assisting in protein folding. Exposed CALR plays a wide range of roles in coordinating innate immune surveillance in physiological and pathological environments [29]. The overall treatment effect of AML adult patients after traditional chemotherapy remains poor, and ultimately affects the prognosis and survival of these patients. Therefore, by mean of public database mining, we next screened anti-tumor targeted drugs based on risk characteristics and some drug sensitivities, so as to help specialist physicians provide potential treatment options with clinical benefits. Our analysis may provide potential treatment strategies for AML patients and theoretical reference for precise treatment of AML. However, the validity of these results still requires further in vitro and in vivo experiments to confirm.

We consider the following advantages of our study. Firstly, Yan et al. [30] constructed a key prognostic gene in the AML immune microenvironment several years ago, but we further calculated the prognostic value of the model we constructed, tumor mutation burden, and explored the tumor microenvironment of different subtypes of ICD. Secondly, compared to other signatures [31–35], our prognostic model (time-dependent ROC curve) (AUC of 0.75, 0.77, and 0.87 for 1 year, 3 years and 5 years, respectively) is more meaningful for the prognosis evaluation of AML patients. But we must be awake to the fact that our research remains inadequate. The results in this study are based on bioinformatics analysis of public databases and still need further validation using multicenter clinical samples and experimental methods.

### Electronic supplementary material

Below is the link to the electronic supplementary material.

Supplementary Material 1

Supplementary Material 2

Supplementary Material 3

Supplementary Material 4

Supplementary Material 5

Supplementary Material 6

Supplementary Material 7

Supplementary Material 8

Supplementary Material 9

Supplementary Material 10

Supplementary Material 11

Supplementary Material 12

Supplementary Material 13

## Data Availability

The data generated and analyzed during this study are described in the following data record: 10.6084/m9.figshare.25532368. The original data for this paper can be found in the GEO database (https://www.ncbi.nlm. nih.gov/geo/), (the accession number is GSE37642) and TCGA database (https://portal.gdc.cancer.gov/).

## Abbreviations

ICD: Immunogenic Cell Death
AML: Acute Myeloid Leukemia
ICDGs: ICD-Related Genes
GEO: Gene Expression Omnibus
DEGs: Differentially Expressed Genes
GO: Gene Ontology
KEGG: Kyoto Encyclopedia of Genes and Genomes
GSEA: Gene Set Enrichment Analysis
CC: Cellular Components
MF: Molecular Functions
TFs: Transcription Factors
DAMPs: Damage-Associated Molecular Patterns

## References

[1] Shimony S, Stahl M, Stone RM (2023). Acute myeloid leukemia: 2023 update on diagnosis, risk-stratification, and management. Am J Hematol.

[2] Bosshard R, O’Reilly K, Ralston S, Chadda S, Cork D (2018). Systematic reviews of economic burden and health-related quality of life in patients with acute myeloid leukemia. Cancer Treat Rev.

[3] Hou HA, Tien HF (2020). Genomic landscape in acute myeloid leukemia and its implications in risk classification and targeted therapies. J Biomed Sci.

[4] Newell LF, Cook RJ (2021).

[5] Kadia TM, Ravandi F, Cortes J, Kantarjian H (2015). Toward Individualized Therapy in Acute myeloid leukemia: a contemporary review. JAMA Oncol.

[6] Döhner H, Wei AH, Löwenberg B (2021). Towards precision medicine for AML. Nat Rev Clin Oncol.

[7] Zhou J, Wang G, Chen Y, Wang H, Hua Y, Cai Z (2019). Immunogenic cell death in cancer therapy: Present and emerging inducers. J Cell Mol Med.

[8] Kroemer G, Galassi C, Zitvogel L, Galluzzi L (2022). Immunogenic cell stress and death. Nat Immunol.

[9] Galluzzi L, Vitale I, Warren S, Adjemian S, Agostinis P, Martinez AB, et al. Consensus guidelines for the definition, detection and interpretation of immunogenic cell death. J Immunother Cancer. 2020;8(1). 10.1136/jitc-2019-000337. 10.1136/jitc-2019-000337 PMC7064135 32209603

[10] Galluzzi L, Kepp O, Hett E, Kroemer G, Marincola FM (2023). Immunogenic cell death in cancer: concept and therapeutic implications. J Transl Med.

[11] Li Z, Lai X, Fu S, Ren L, Cai H, Zhang H (2022). Immunogenic cell death activates the Tumor Immune Microenvironment to Boost the Immunotherapy Efficiency. Adv Sci (Weinh).

[12] Lilljebjörn H, Orsmark-Pietras C, Mitelman F, Hagström-Andersson A, Fioretos T (2022). Transcriptomics paving the way for improved diagnostics and precision medicine of acute leukemia. Semin Cancer Biol.

[13] Bazinet A, Kantarjian HM (2023). Moving toward individualized target-based therapies in acute myeloid leukemia. Ann Oncol.

[14] Garg AD, De Ruysscher D, Agostinis P (2016). Immunological metagene signatures derived from immunogenic cancer cell death associate with improved survival of patients with lung, breast or ovarian malignancies: a large-scale meta-analysis. Oncoimmunology.

[15] Serroukh Y, Hébert J, Busque L, Mercier F, Rudd CE, Assouline S (2023). Blasts in context: the impact of the immune environment on acute myeloid leukemia prognosis and treatment. Blood Rev.

[16] Galluzzi L, Buqué A, Kepp O, Zitvogel L, Kroemer G (2017). Immunogenic cell death in cancer and infectious disease. Nat Rev Immunol.

[17] Gaglia G, Burger ML, Ritch CC, Rammos D, Dai Y, Crossland GE (2023). Lymphocyte networks are dynamic cellular communities in the immunoregulatory landscape of lung adenocarcinoma. Cancer Cell.

[18] Maskalenko NA, Zhigarev D, Campbell KS (2022). Harnessing natural killer cells for cancer immunotherapy: dispatching the first responders. Nat Rev Drug Discov.

[19] Wolf NK, Kissiov DU, Raulet DH (2023). Roles of natural killer cells in immunity to cancer, and applications to immunotherapy. Nat Rev Immunol.

[20] Im AP, Sehgal AR, Carroll MP, Smith BD, Tefferi A, Johnson DE (2014). DNMT3A and IDH mutations in acute myeloid leukemia and other myeloid malignancies: associations with prognosis and potential treatment strategies. Leukemia.

[21] Daver N, Venugopal S, Ravandi F (2021). FLT3 mutated acute myeloid leukemia: 2021 treatment algorithm. Blood Cancer J.

[22] Daver N, Schlenk RF, Russell NH, Levis MJ (2019). Targeting FLT3 mutations in AML: review of current knowledge and evidence. Leukemia.

[23] Gonzales F, Barthélémy A, Peyrouze P, Fenwarth L, Preudhomme C, Duployez N, Cheok MH (2021). Targeting RUNX1 in acute myeloid leukemia: preclinical innovations and therapeutic implications. Expert Opin Ther Targets.

[24] Falini B, Brunetti L, Sportoletti P, Martelli MP (2020). NPM1-mutated acute myeloid leukemia: from bench to bedside. Blood.

[25] Zhou X, Zhou S, Li B, Li Q, Gao L, Li D (2015). Transmembrane TNF-α preferentially expressed by leukemia stem cells and blasts is a potent target for antibody therapy. Blood.

[26] Moreno Ayala MA, Campbell TF, Zhang C, Dahan N, Bockman A, Prakash V (2023). CXCR3 expression in regulatory T cells drives interactions with type I dendritic cells in tumors to restrict CD8(+) T cell antitumor immunity. Immunity.

[27] Speiser DE, Chijioke O, Schaeuble K, Münz C (2023). CD4(+) T cells in cancer. Nat Cancer.

[28] Madsen RR, Vanhaesebroeck B, Semple RK, Cancer-Associated (2018). PIK3CA mutations in Overgrowth disorders. Trends Mol Med.

[29] Sen Santara S, Lee DJ, Crespo Â, Hu JJ, Walker C, Ma X (2023). The NK cell receptor NKp46 recognizes ecto-calreticulin on ER-stressed cells. Nature.

[30] Yan H, Qu J, Cao W, Liu Y, Zheng G, Zhang E (2019). Identification of prognostic genes in the acute myeloid leukemia immune microenvironment based on TCGA data analysis. Cancer Immunol Immunother.

[31] Xie JY, Wang WJ, Wang N, Dong Q, Han H, Feng YP (2023). A novel immune-related gene signature correlated with serum IL33 expression in acute myeloid leukemia prognosis. Am J Transl Res.

[32] Wei Y, Miao Z, Guo X, Feng S (2023). Exploration of cuprotosis-related genes for predicting prognosis and immunological characteristics in acute myeloid leukaemia based on genome and transcriptome. Aging.

[33] Yang Y, Yang Y, Liu J, Zeng Y, Guo Q, Guo J (2022). Establishment and validation of a carbohydrate metabolism-related gene signature for prognostic model and immune response in acute myeloid leukemia. Front Immunol.

[34] Rong D, Chen X, Xiao J, Liu D, Ni X, Tong X (2022). Histone methylation modification patterns and relevant M-RiskScore in acute myeloid leukemia. Heliyon.

[35] Han C, Zheng J, Li F, Guo W, Cai C (2022). Novel prognostic signature for Acute myeloid leukemia. Bioinformatics Analysis of Combined CNV-Driven and ferroptosis-related genes. Front Genet.

