# Supplementary material for: Comprehensive characterization of immunogenic cell death in acute myeloid leukemia revealing the association with prognosis and tumor immune microenvironment
Source: BMC Med Genomics. 2024 Apr 26;17:107. doi: 10.1186/s12920-024-01876-w (PMC11046942; doi:10.1186/s12920-024-01876-w)
Supplement: Supplementary file 12 — Supplementary Material 12 [file 12920_2024_1876_MOESM12_ESM.docx]

Additional file 1: AML exp in TCGA database

Additional file 2: Related genes and immune cell expression matrix

Additional file 3: Clinical information

Additional file 4: Significantly deferentially expressed genes

Additional file 5: GSEA analysis outcome

Additional file 6: Immune infiltration data in training and validation sets

Additional file 7: The relationship between gene expression and prognosis score

Additional file 8: Gene-miRNA results

Additional file: 9 Gene-TF results

Additional file: 10 Drug response data in drug sensitivity analysis

Additional file: 11 Gene expression data in drug sensitivity analysis

Additional file: 12 Related results of drug sensitivity

Additional file: 13.Data Index
